# Gut and skin microbiota of *Bufo gargarizans* tadpoles respond differently to temperature

**DOI:** 10.3389/fmicb.2026.1835806

**Published:** 2026-05-05

**Authors:** Liqun Ma, Fangnan Jin, Xiaoyu Guo, Cong Han, Muhammad Irfan, Zhibing Li, Lixia Zhang

**Affiliations:** 1 Department of Ecology, College of Life Sciences, Henan Normal University, Xinxiang, China; 2 Department of Biotechnology, University of Sargodha, Sargodha, Pakistan; 3 Puyang Field Scientific Observation and Research Station for Yellow River Wetland Ecosystem, Henan, China

**Keywords:** *Bufo gargarizans*, development, gut microbiota, skin microbiota, temperature

## Abstract

Gut and skin microbiota impact host physiology and can be influenced by environmental changes, however, their reactions to rising environmental temperatures remain unclear. In this study, *Bufo gargarizans* tadpoles were exposed to 22 °C (control) and 27 °C (high-temperature) and the gut and skin microbiota were sampled at 24, 72, and 144 h to assess the effects of short-term elevated temperatures. Results revealed that the richness index (ACE, Chao, Sobs) and Pd index of gut microbiota were significantly affected by stress time, while the diversity index (Shannon, Simpson) was significantly affected by the interaction of temperature and time. The response of alpha diversity of skin microbiota to temperature and time was not significant, and only Pd index was affected by the interaction between temperature and time. No significant changes in microbial composition (beta diversity) were observed in either skin or gut microbiota across three sampling time points under both treatments. After 144 h of high-temperature stress, KEGG functional prediction indicated that basic metabolic pathways involving DNA replication and amino acid biosynthesis were significantly downregulated in the gut microbiota, while sulfur metabolism was upregulated. For the skin microbiota, pathways related to drug resistance, cancer, and metabolism showed significantly decreased abundance, whereas homologous recombination and metabolic pathways were significantly elevated. High temperature accelerated the developmental rate of tadpoles, however, their growth metrics including weight, total length, snout-vent length, and body width were significantly inhibited at equivalent developmental stages. This study elucidates the response patterns of amphibian hosts and their commensal microbiota to short-term high-temperature stress, providing insights into the implications of global climate change for amphibian survival.

## Introduction

Global climate change has become a central topic of concern in contemporary ecology and conservation biology. The Intergovernmental Panel on Climate Change (IPCC) projects a continued rise in global mean surface temperatures over the coming decades, accompanied by increases in the frequency and intensity of extreme weather events (e.g., heatwaves), thereby profoundly affecting ecosystem stability as well as the survival and reproduction of species (Rohr and Palmer, 2013; Ayanlade et al., 2020; Ujszegi et al., 2022; Murali et al., 2023). As typical ectothermic vertebrates, amphibians have limited capacity for thermoregulation, and their physiological, metabolic, and behavioral processes are strongly constrained by ambient temperature; consequently, they are widely regarded as one of the vertebrate taxa most severely threatened by ongoing climate warming (Padilla et al., 2019; Bofill and Blom, 2024; Gross, 2025). Heat stress not only directly affects amphibian metabolic rates, metamorphic development, and survival, but may also influence host health indirectly by reshaping their symbiotic microbial communities (Jani, 2019; Li et al., 2020; Niu et al., 2023; Xu et al., 2024).

The symbiotic microbiota of amphibians is closely linked to host health, pathogen resistance, immune regulation, and adaptation to environmental change (Bletz et al., 2017; Zhu et al., 2024). The gut microbiota primarily contributes to nutrient acquisition, energy metabolism, and host growth and development (Tremaroli and Bäckhed, 2012). As the first immune barrier of amphibians, the skin microbiota secretes antimicrobial peptides and antifungal metabolites, which are of great significance for inhibiting the growth of pathogens and maintaining skin immune homeostasis (Harris et al., 2009; Chehoud et al., 2013; Sanford and Gallo, 2013; Nakatsuji et al., 2017; Nguyen and Soulika, 2019; Wang et al., 2021). A comprehensive understanding of a host’s immunological capacity and general well-being can be achieved by analyzing the composition and diversity of its associated microbiomes.

The composition of amphibian symbiotic microbiomes is highly susceptible to temperature variations, largely due to the diverse thermal sensitivities among different microbial taxa (Wang et al., 2025). As a critical ecological factor, ambient temperature has significant influence on the structure and function of amphibian gut microbiota (Huang and Liao, 2021; Wang et al., 2025). High-temperature stress often leads to a decrease in the diversity of gut microbiota and an increase in individual differences, thus breaking the homeostasis state of microbial communities (Kohl and Yahn, 2016). Under the condition of elevated temperatures, the relative abundance of disease-resistant bacterial taxa decreases, while that of potentially pathogenic taxa increases (Fontaine et al., 2018). Changes in gut microbiota composition can cause intestinal disease, infection, and immune impairment, so this may reduce the survival possibility of hosts (Bestion et al., 2017). Temperature fluctuations not only can directly cause seasonal changes inside microbial communities, but also can indirectly adjust the number of microbes by changing host physiological characteristics, such as skin sloughing frequency (Meyer et al., 2012; Ohmer et al., 2014; Longo et al., 2015; Sun et al., 2025). In addition, temperature fluctuations may alter the bioactivity of antimicrobial compounds derived from skin microbiota, which influencing host immune defenses and overall health (Daskin et al., 2014; Woodhams et al., 2014).

*Bufo gargarizans* (Amphibia: Anura: Bufonidae: *Bufo*) is a widely distributed toad in East Asia. It possesses both ecological and medicinal value and is highly sensitive to environmental changes, making it an ideal model for studying thermal adaptation and climate warming in amphibians (Nalbantsoy et al., 2016; Jiang et al., 2017; Pu et al., 2021; Meng et al., 2024). An increasing body of evidence indicates that elevated thermal stress accelerates metabolic rates in amphibian tadpoles, potentially leading to developmental malformations, reduced survival rates, and altered behavioral strategies (Tejedo et al., 2010; Bellakhal et al., 2014; Ruthsatz et al., 2018). For instance, high temperature significantly decreased the total length, snout-vent length, and hindlimb length, and suppressed skeletal development, in *Rana chensinensis* tadpoles at stage Gs42 (Niu et al., 2023). High temperature (28 °C) accelerated development but reduced body size and weight in *B. gargarizans* tadpoles, while low temperature (16 °C) inhibited development and increased body size and weight (Chen et al., 2021). However, current research has mostly focused on the immediate outcomes of high temperatures upon tadpoles’ morphology and physiological indicators, while the inner microbiological mechanisms, especially the effect on symbiotic microbial groups, have not been sufficiently studied.

In this study, we experimentally addressed two key questions by exposing *B. gargarizans* tadpoles to control and high-temperature: (1) How does high temperature change the composition of both gut and skin microbiota? (2) How does short-term high-temperature affect tadpole growth and development? We will analyze the response mechanisms of amphibians to climate warming from the perspective of “host-microbe” interactions. The findings will provide a theoretical basis for assessing population conservation strategies for amphibians under extreme climate conditions.

## Materials and methods

### Animal husbandry

In April 2023, 300 wild *B. gargarizans* tadpoles at Gosner stage 30 were collected from the Huanglian Forest Farm (112°07′53″E, 35°15′15″N) in Jiyuan City, Henan Province, China. The tadpoles were arbitrarily separated into six distinct groups. Each group was reared in a separate 10 L polycarbonate tank containing dechlorinated tap water. An aquarium water-heating apparatus was used to maintain the water temperature at 22 °C. A lighting schedule consisting of 12 h of illumination followed by 12 h of darkness was put into effect. During a seven-day acclimatization period, autoclaved artificial food was given to the tadpoles every day at 8:00 o’clock. The feeding quantity was equal to 8% of their body weight. A daily 30% water change was performed by using dechlorinated water that had been pre-heated to the experimental temperature.

Following this 7 day acclimation, three tanks were randomly assigned as the high-temperature treatment group. Water temperature was gradually raised from 22 °C to 27 °C within 75 min using a heating device (increasing 1 °C every 15 min). The remaining three tanks served as the control group, maintaining a constant water temperature of 22 °C. Control temperature (22 °C) was based on the local average aquatic environmental temperature measured *in situ* (22.3 °C). The 27 °C high temperature treatment was selected to simulate end of century climate warming, as the SSP3-7.0 scenario projects regional summer temperature increases of approximately 4–5 °C above baseline (Tebaldi et al., 2021). Moreover, prior research has indicated that even minor temperature disparities, such as a 5 °C difference, can significantly influence the gut microbiota of animals (Fontaine and Kohl, 2020). During the experiment, the water temperature in each group was continuously monitored to ensure that the actual temperature fluctuation range was controlled within ±0.5 °C of the set value. We conducted all animal experiments strictly adhering to national standards for laboratory animal welfare, following protocol approval by the Institutional Animal Care and Use Committee of Henan Normal University.

### Sample collection and sequencing

On days 1, 3, and 6 (i.e., 24, 72, and 144 h) post the onset of temperature elevation, four tadpoles were selected at random from each tank and euthanized humanely with MS-222. Gut and skin microbiota were aseptically collected. Total genomic DNA of the microbial communities was extracted using the MagAttract PowerSoil Pro DNA Kit (Qiagen, Hilden, Germany). The recovered nucleic acids were subjected to 2% agarose gel electrophoresis for integrity confirmation and analyzed via a NanoDrop 2000 (Thermo Fisher Scientific, United States) to determine concentration and purity. PCR was employed to selectively amplify the V3-V4 subregions of the 16S ribosomal RNA gene, using the forward primer 338F (5′-ACTCCTACGGGAGGCAGCAG-3′) and the reverse primer 806R (5′-GGACTACHVGGGTWTCTAAT-3′). The PCR procedure and parameters are detailed in Supplementary material (Part 3). High-throughput microbial profiling was performed at Majorbio Bio-Pharm Technology Co., Ltd. (Shanghai, China) using the Illumina MiSeq sequencing system.

### Statistical analysis

To validate the comprehensiveness of our sampling effort, rarefaction curves were plotted within the R environment (v3.3.1). This analysis utilized OTUs aggregated at a ≥ 97% identity cutoff via Mothur (v1.30.2), and the observation that these curves flattened out indicated adequate community coverage.

We investigated how the alpha diversity of gut and skin microbiota responded to temperature, time, and their interaction by constructing Linear Mixed-Effects Models (LMMs) using JMP Pro 17 software.

Variations in microbial community structures were statistically validated through a PERMANOVA approach applied to Bray-Curtis metrics (QIIME v1.9.1). Statistical significance was robustly determined via 999 permutations, with an FDR correction applied to mitigate multiple testing errors. Significant temporal changes triggered by thermal stress were initially screened using the R-based adonis command. To evaluate the variations across varying thermal treatments and time points, pairwise PERMANOVA was executed utilizing three distinct metrics: Bray-Curtis dissimilarities alongside both weighted and unweighted UniFrac matrices. The spatial separation of these microbial profiles was graphically represented via Principal Coordinate Analysis (PCoA).

For the dominant bacterial phylum or genus with an average relative abundance exceeding 1% in gut and skin samples, we performed Analysis of Covariance (ANCOVA) using the “Response Screening” module of JMP Pro 17. Before the model was constructed, the relative abundance data was subjected to arcsine square root conversion to optimize its data distribution. The converted abundance was used as a response variable, and temperature, time and their interactions were included in the model as predictors. The FDR correction was applied to all *p*-values, which are denoted as q-values.

To infer the functional potential of the gut and skin microbiota, PICRUSt2 was employed to analyze the 16S rRNA sequencing data collected at the 144-h mark. Metagenomic functional predictions were executed by aligning the OTU profiles against the Kyoto Encyclopedia of Genes and Genomes (KEGG) database. Afterwards, we identified metabolic pathways significantly influenced by thermal stress using Welch’s t-test within the R environment.

### Morphometric measurement

Sampling was conducted 144 h after the experiment began, around 10 tadpoles were chosen at random from each tank. After euthanasia with MS-222, specimens were fixed in 4% paraformaldehyde for 24 h and subsequently preserved in 70% ethanol (Niu et al., 2023). A total of 31 tadpoles were sampled from the control group (22 °C) and 30 from the high-temperature group (27 °C). Developmental stages were determined based on Gosner’s system (Gosner, 1960). Morphometric data were recorded, the weight was determined with an analytical balance (accurate to 0.001 g), and the linear dimensions including total length, snout-vent length (SVL), body width, body height, and tail length were quantified using a digital Vernier caliper with a 0.001 mm resolution.

## Results

### Diversity analysis

A total of 1,238,055 and 1,376,478 valid sequences were obtained from the gut and skin samples of *B. gargarizans* tadpoles, with an average of 76,471 and 68,781 sequences, respectively. After clustering according to the 97% similarity standard, the gut and skin microbiota contained 1,114 and 879 OTUs, respectively. The rarefaction analysis (Supplementary Figure S1) demonstrated that species richness approached an asymptote in all groups, confirming that sequencing depth provided a comprehensive representation of the microbial community composition.

**Figure 1:**
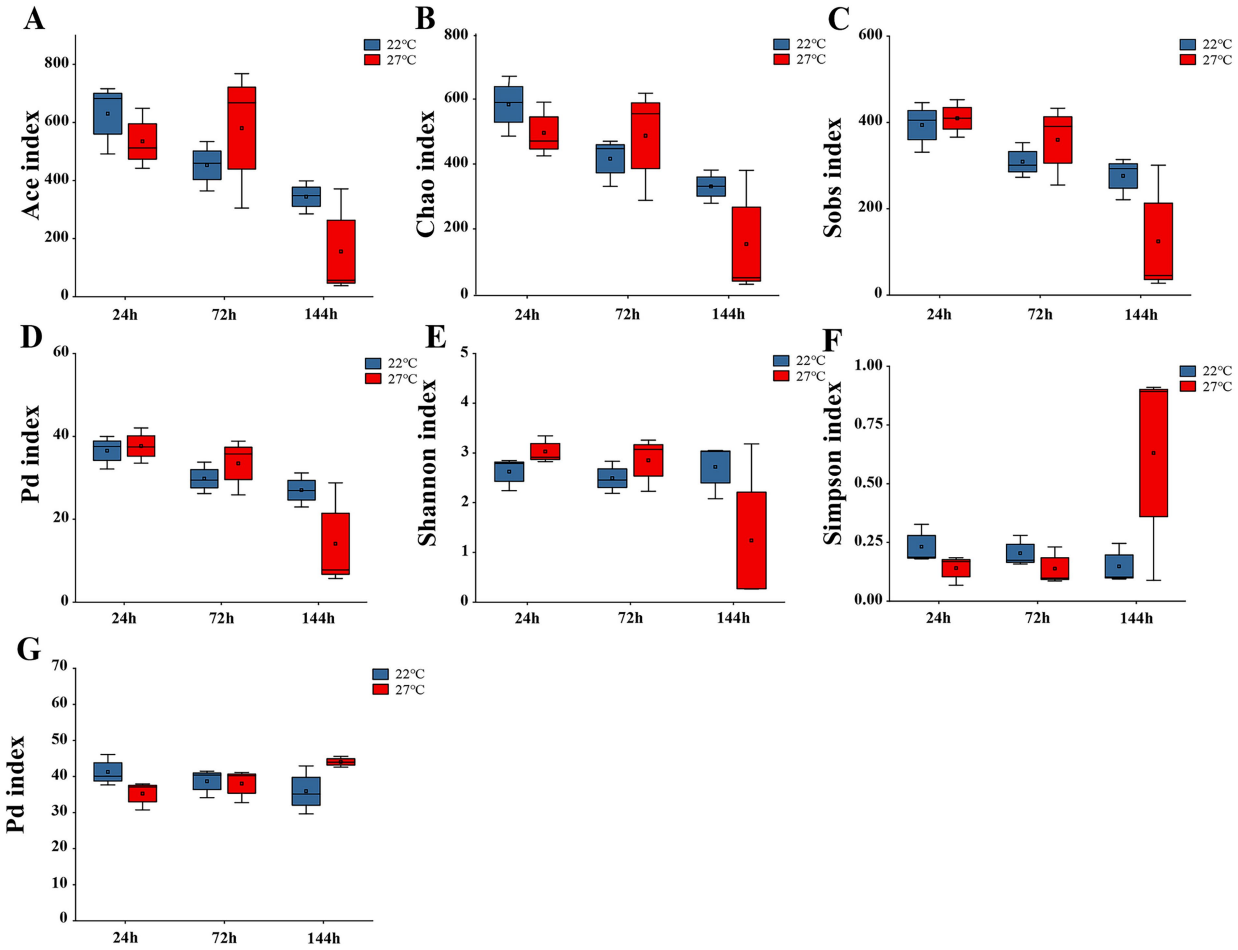
Alpha diversity of gut and skin microbiota in *B. gargarizans* tadpoles under two temperatures and three time points. The bold center line represents the median, the box length represents the IQR, and the whiskers extend to 1.5 times the interquartile range (IQR). Points beyond this range are plotted individually. **(A–D)** The Ace, Chao, Sobs, and Pd indices of the gut microbiota were significantly influenced by incubation time (LMMs, *p* < 0.01), but not by temperature. **(E–F)** The Shannon and Simpson indices of the gut microbiota were significantly affected by the interaction between temperature and time (LMMs, *p* < 0.05), with no significant effect of temperature. **(G)** The Pd index of the skin microbiota was significantly affected by the interaction between temperature and time (LMMs, *p* < 0.05), while neither temperature nor time alone had a significant effect on the remaining alpha diversity indices.

Results from the linear mixed models (LMMs) indicated that gut microbial alpha diversity remained stable across different temperatures, whereas a notable temporal shift was observed in the Ace, Chao, Sobs, and Pd indices (Figures 1A–D; Supplementary Table S1, LMMs, *p* < 0.01), these indices significantly decreased over time under the experimental conditions. Moreover, the Shannon and Simpson indices were modulated by a significant interplay between temperature and time (Figures 1E,F; Supplementary Table S1, LMMs, *p* < 0.05). In the high-temperature group, the Shannon index significantly decreased, whereas the Simpson index significantly increased over time. The alpha diversity index of the skin microbiota was not affected by temperature and time alone (Ace, Chao, Shannon, Simpson, Sobs, and Pd). However, the interaction between temperature and time exerted a significant influence on the Pd index (Figure 1G; Supplementary Table S2, LMMs, *p* < 0.05), under high-temperature conditions, the Pd index of skin microbiota increased significantly with time.

To investigate variations in microbial community composition, PERMANOVA was performed based on Bray–Curtis dissimilarity matrices. The results highlighted niche-specific responses to environmental variables, where the gut microbial configuration was significantly shaped by both temperature (Supplementary Table S3, PERMANOVA, FDR *p* < 0.05) and time (Supplementary Table S3, PERMANOVA, FDR *p* < 0.01), whereas skin microbial communities were primarily driven by time (Supplementary Table S3, PERMANOVA, FDR *p* < 0.01) and remained largely insensitive to temperature fluctuations. We performed pairwise PERMANOVA to compare the two temperature groups at intervals of 24 h, 72 h, and 144 h. Using a combination of Bray-Curtis, unweighted UniFrac, and weighted UniFrac metrics. Regardless of the sampling time point, the microbial community structures of both the gut and skin remained statistically indistinguishable between the two temperature regimens, as demonstrated by the PCoA plots (Supplementary Figures S2, S3, all *p* > 0.05).

### Differential species analysis

After filtering out rare taxa (average relative abundance <1%), the gut microbiota retained 5 phyla and 15 genera, the skin microbiota retained 5 phyla and 14 genera. ANCOVA analysis revealed that time had a significant effect on gut microbiota composition, particularly on 3 phyla and 7 genera (Table 1, FDR *p* < 0.05). Among them, the relative abundances of Fusobacteria, Firmicutes, *Acetobacter*, *Cetobacterium*, *Tyzzerella*, *unclassified-f-Gracilibacteraceae*, *unclassified-f-Ruminococcaceae* and *unclassified-f-Lachnospiraceae* significantly decreased over time. Conversely, relative abundances of Proteobacteria and *unclassified-f-Enterobacteriaceae* rose significantly as time progressed. Temperature significantly influenced the relative abundances of 2 phyla and 1 genus (Table 1, FDR *p* < 0.05), Actinobacteria and *Acetobacter* exhibited higher relative abundances under control temperature conditions, while Proteobacteria showed higher relative abundance under high-temperature conditions. Furthermore, a significant interaction between temperature and time was observed for the relative abundances of 2 phyla and 2 genera (Table 1, FDR *p* < 0.05). Under high-temperature conditions, the relative abundances of Firmicutes and *unclassified-f-Lachnospiraceae* declined significantly over time, whereas those of Proteobacteria and *unclassified-f-Enterobacteriaceae* significantly increased. The genera *Aeromonas* and *Limnohabitans* in the skin microbiota were significantly affected by time (Table 1, FDR *p* < 0.05). Specifically, a significant temporal decrease in the relative abundance of *Aeromonas* was observed, while *Limnohabitans* displayed a significant increase over time. No significant effects of temperature or the temperature and time interaction were detected on the relative abundances of individual phyla or genera in the skin microbiota.

**Table 1:** Taxonomic groups in gut and skin microbiota with significant abundance variations driven by temperature, time, and their interaction.

Location	Species	Time	Temp	Time*Temp	*F* statistic	*q*-value
Gut	Phylum					
	Firmicutes	−			54.392	<0.01
	Fusobacteriota	−			14.287	<0.05
	Proteobacteria	+			69.533	<0.01
	Actinobacteriota		C		6.725	<0.05
	Proteobacteria		W		9.148	<0.05
	Firmicutes			↓(W)	8.714	<0.05
	Proteobacteria			↑(W)	11.432	<0.05
	Genera					
	*Acetobacterium*	−			25.439	<0.01
	*Cetobacterium*	−			14.233	<0.05
	*Tyzzerella*	−			20.058	<0.01
	*unclassified_f__Enterobacteriaceae*	+			9.83	<0.05
	*unclassified_f__Gracilibacteraceae*	−			21.308	<0.01
	*unclassified_f__Lachnospiraceae*	−			13.977	<0.05
	*unclassified_f__Ruminococcaceae*	−			26.308	<0.01
	*Acetobacterium*		C		10.672	<0.05
	*unclassified_f__Enterobacteriaceae*			↑(W)	9.882	<0.05
	*unclassified_f__Lachnospiraceae*			↓(W)	9.838	<0.05
Skin	Genera					
	*Aeromonas*	−			13.998	<0.05
	*Limnohabitans*	+			21.184	<0.05

Time: Temporal changes in abundance are marked by (+) for increases and (−) for decreases. Temp: Temperature effects are indicated as (W) for greater abundance in the high-temperature (Warm) cohort, and (C) for greater abundance in the Control group. Time × Temp: Represents the interaction effect; arrows illustrate the direction of the temporal trend, while (W) or (C) specifies the temperature condition where this trend is observed. Statistics: *F*-statistics and FDR-adjusted *p*-values (*q*-values) were derived using ANCOVA.

### Functional prediction

**Figure 2:**
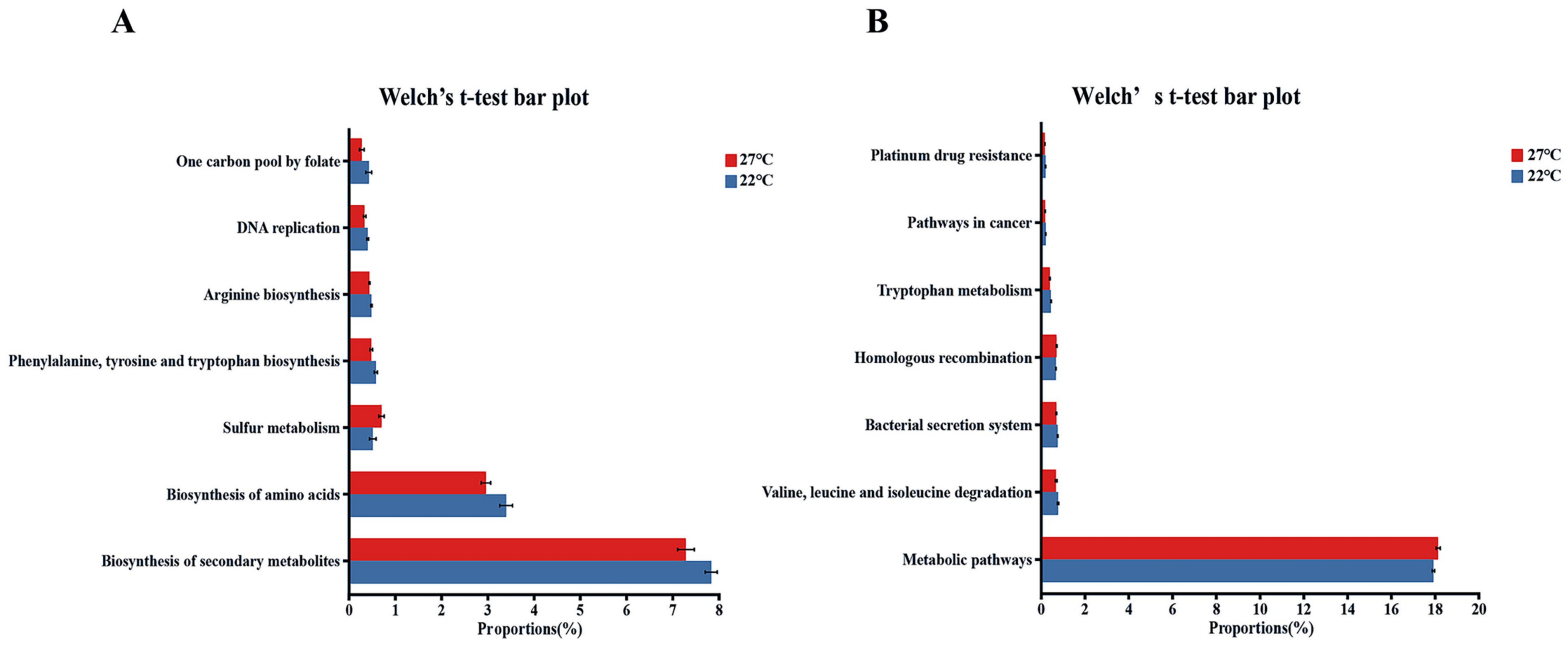
Significant shifts in KEGG metabolic pathways within the gut and skin microbiota of *B. gargarizans* tadpoles following 144 h of temperature exposure. Functional profiles were predicted via PICRUSt2, and the statistical significance of differences in relative abundance between temperature groups was evaluated using Welch’s two-sample *t*-test in R. **(A)** KEGG functional pathway map of the gut microbiota; **(B)** KEGG functional pathway map of the skin microbiota.

Functional prediction using PICRUSt2 showed significant alterations in microbial functional abundances in the high-temperature treatment relative to the control group. In the gut samples, high temperature significantly suppressed six biosynthesis related pathways, such as DNA replication, Biosynthesis of amino acids, and One carbon pool by folate, while simultaneously boosting the relative abundance of the Sulfur metabolism pathway (Figure 2A, *p* < 0.05). The skin samples indicated a significant downregulation of five pathways, including Platinum drug resistance and Bacterial secretion system, whereas homologous recombination and metabolic pathways were significantly enriched (Figure 2B, *p* < 0.05).

### Developmental responses of *B. gargarizans* tadpoles to short-term high-temperature stress

**Figure 3:**
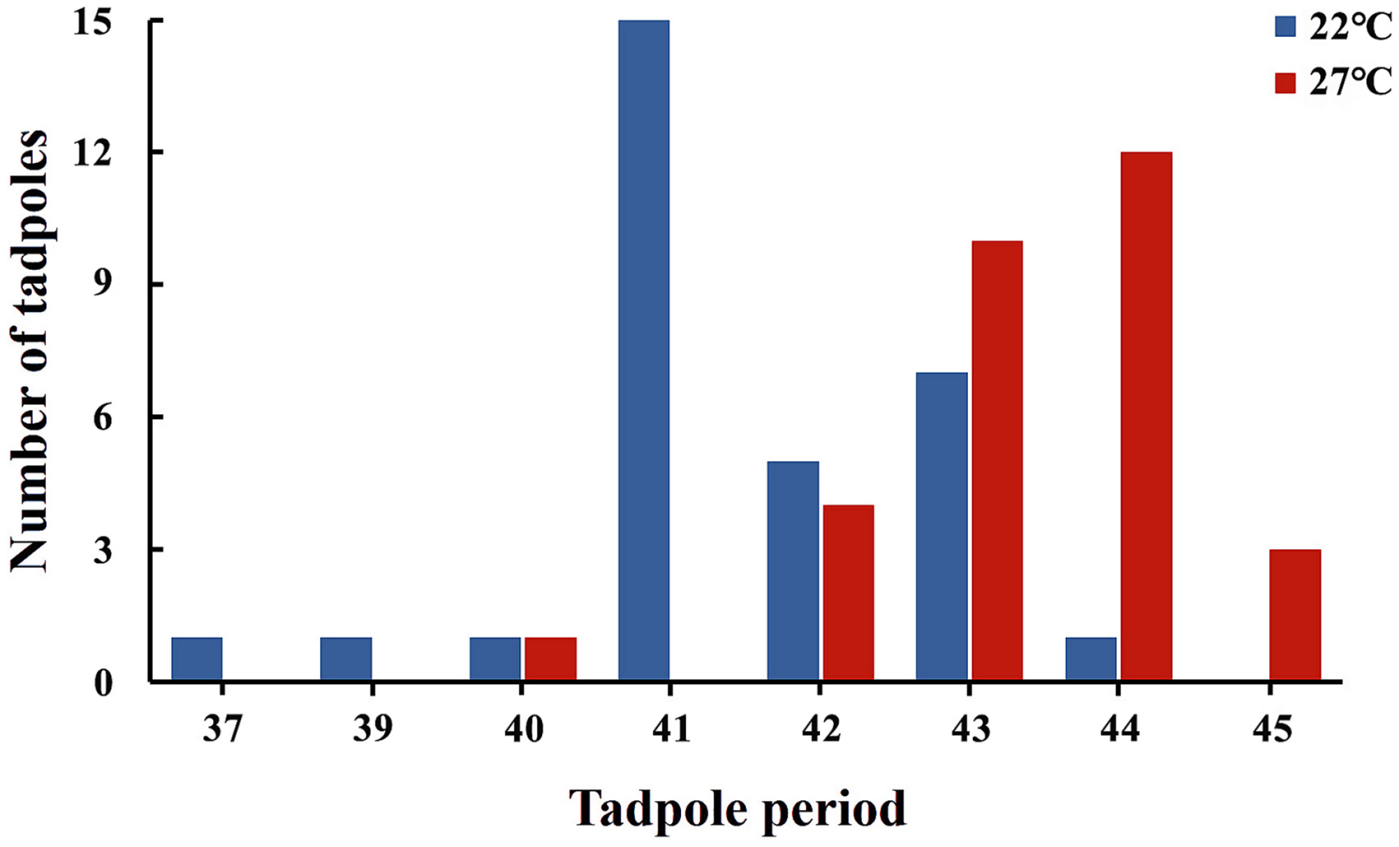
Developmental stages of *B. gargarizans* tadpoles after 144 h under two temperature treatments. Tadpoles in the control (22 °C) group reached Gs41, while those in the high-temperature (27 °C) group reached Gs44 (Mann–Whitney U test, z = −5.084, *p* < 0.01). Shapiro–Wilk tests indicated that the data from both groups were non-normally distributed (control group: *n* = 31; high-temperature group: *n* = 30). The Mann–Whitney U test was used to analyze differences in developmental stages between temperature treatments.

Morphological analysis revealed that tadpoles reared under high-temperature conditions developed significantly more rapidly than those in the control group. Specifically, tadpoles in the control group were at Gs41, whereas those in the high-temperature group had reached Gs44 (Figure 3, *z* = −5.084, *p* < 0.01). Relative to the control group, Gs42 tadpoles exposed to high-temperature treatment exhibited significant decreases in weight, total length, SVL, and body width, while body height and tail length were not significantly affected (Supplementary Figure S4; Supplementary Table S4, *p* < 0.05). Similarly, at Gs43, significant decreases were observed in weight, total length, SVL, body width, and tail length in the high-temperature group, while no significant variation was detected in body height (Supplementary Figure S5; Supplementary Table S5, *p* < 0.01).

## Discussion

### High temperature stress accelerates developmental rate in tadpoles

Amphibians have the characteristics of high skin permeability and complex life cycle. Their larvae rely on aquatic environment to complete metamorphosis, while adults live in amphibious environment. Amphibians are extremely sensitive to environmental fluctuations, particularly temperature changes (Niu et al., 2023; Shadle et al., 2023), temperature is widely recognized as a major factor governing metamorphosis and body size in anuran amphibians (Dastansara et al., 2017; Ruthsatz et al., 2018). We observed that high-temperature treatment resulted in a significantly accelerated developmental rate in tadpoles compared with the control group. This result is consistent with other published research conclusions (Tejedo et al., 2010; Chen et al., 2021), which is consistent with the Temperature-Size Rule (TSR) in amphibian ecology. This rule predicts that the ectotherms will develop faster at higher temperatures, but the adult size will be smaller (Verberk et al., 2021; Zhu et al., 2023).

### Shifts in gut microbiota composition under high temperature stress

During long-time evolution process, the gut microbiota has built a mutually helpful symbiotic connection with its host, and it plays a very important regulatory function for maintaining the host’s environmental homeostasis (Sommer and Bäckhed, 2013; Shortt et al., 2018). Existing study documents show that, during amphibian metamorphosis, the gut microbiota has big changes in its composition (Kohl et al., 2013; Chai et al., 2018; Tong et al., 2020). For instance, the metamorphosis of *R. chensinensis* tadpoles is followed by deep changes in both microbial diversity and its composition (Yang et al., 2020). From larvae to adult stages, the diversity and number of gut microbiota of *Andrias davidianus* have a gradual reduction (Cai et al., 2023). In this research, the number of beneficial bacteria like Actinobacteria and Firmicutes went down, while the number of conditional pathogenic bacteria such as Proteobacteria went up. It may be that high temperature treatment leads to changes in the intestinal environment of tadpoles, creating an intestinal environment that is more conducive to the proliferation of potential pathogenic bacteria and is not conducive to the survival of some beneficial bacteria. Some members belonging to the Firmicutes phylum have the ability to digest dietary fiber and change it into volatile fatty acids (VFAs). These metabolites take part in controlling the metabolism of nutrient absorption and energy storage, thereby promoting the growth and development of the host (Sun et al., 2023; Zhang et al., 2024), Therefore, the high-temperature reduction of Firmicutes and the consequent decrease in VFAs may limit the energy supply required for host growth, leading to smaller body size during development and adversely affecting host health. The increased abundance of Proteobacteria is a potential diagnostic marker of gut microbiota imbalance and disease risk (Shin et al., 2015). Studies have demonstrated that the number of Proteobacteria has a negative correlation with antioxidant enzyme activity. People guess that the increase of Proteobacteria may cause inflammation and oxidative stress through destroying the function of intestinal barrier (Jin et al., 2024; Xie et al., 2025). In addition, temperature changes can lead to an increase in the abundance of intestinal pathogens and changes in the expression of lipid metabolism genes, which then lead to intestinal diseases, bacterial infections and immunodeficiency, and finally increase the health risk and immune burden of the host (Bestion et al., 2017; Niu et al., 2023).

### High temperature stress promotes skin immune barrier maturation and alters skin microbiota

The results demonstrated that high temperature stress promotes a more rapid developmental rate in tadpoles, promoted the development of skin mucus glands and enhanced the ability of antimicrobial peptide synthesis, thus forming a more perfect innate immune barrier (Barak et al., 2005; Torres et al., 2026). As a common opportunistic pathogen in amphibians, *Aeromonas* faces elevated survival pressure and experiences reduction of its relative abundance in the course of skin immune system maturation. This change is consistent with the general law of succession of host symbiotic microorganisms with the maturation of the immune system during individual development (Bataille et al., 2018). Kueneman et al. (2014) also reported a similar phenomenon that the skin microbiota structure of *Rana cascadae* tadpoles was reorganized after metamorphosis, accompanied by changes in immune function. Mucus secreted by amphibian skin mucous glands serves as a vital substrate for symbiotic microorganisms (Woodhams et al., 2014; Brunetti et al., 2023; Woodhams et al., 2023), availability of this substrate is likely a key determinant contributing to the elevated relative abundance of *Limnohabitans*.

### Functional alterations in microbiota induced by high temperature

Functional profiling by means of PICRUSt2 showed that high-temperature stress produced significant alteration on functional landscape of gut and skin microbiota in *B. gargarizans* tadpoles. Folate metabolism is a core route for one-carbon group transfer, taking part in DNA synthesis, amino acid metabolism, and methylation reactions. High temperatures might suppress predicted folate synthesis genes (e.g., *folP*, *folK*) inside microbiota, potentially disrupting the steady state of host one-carbon metabolism and weakening cell proliferation as well as neurotransmitter synthesis (Li et al., 2023). The observed suppression of DNA replication pathway could be related to oxidative stress caused by high temperatures, which may harm microbial DNA (Slimen et al., 2014), or might indicate direct inhibition on microbial proliferation, potentially contributing to the reduced gut microbiota diversity in tadpoles. Arginine is a very important material for keeping immune function and intestinal barrier. High-temperature stress may inhibit arginine synthesis genes (e.g., *ARG*) in microbiota, potentially lowering arginine supply and thus possibly weakening tadpoles’ anti-infection ability become weaker (Agus et al., 2018). Similarly, the predicted production of phenylalanine, tyrosine, and tryptophan, which act as precursors of neurotransmitters, appeared to be restrained under high-temperature conditions. This inhibition may damage tadpoles’ neuroendocrine function, possibly causing behavioral abnormalities or making intestinal inflammation more serious (Blachier et al., 2021). Furthermore, because tadpoles cannot regulate their own body temperature, high surrounding temperatures may directly suppress digestive enzyme activity, reducing nutrient absorption and further affecting microbial amino acid production. On the contrary, the up-regulation of sulfur metabolism may have relation with energy generation; some gut microbes can use sulfides to carry out anaerobic respiration, which could confer a survival advantage in environments with limited energy. However, it is necessary to point out that high concentrations of hydrogen sulfide (H_2_S) can damage the completeness of host intestinal epithelial mucus layer and have connection with intestinal inflammation (Cabrera-Guzmán et al., 2013). In conclusion, high-temperature stress potentially disturbs intestinal functional steady state by inhibiting beneficial anabolic metabolism and inducing possibly harmful energy metabolism in gut microbiota. This disturbance might ultimately leads to weakened nutrient absorption, damaged barrier function, and impaired immune regulation in *B. gargarizans* tadpoles.

Tryptophan metabolism can produce various bioactive metabolites, for instance kynurenine and indole derivatives; these substances have pivotal function in regulation of host immune and barrier functions. Our research results suggest that high temperature may have suppression effect on tryptophan metabolism and degradation of valine, leucine, and isoleucine. Therefore, this suppression could lead to reduced levels of host immunomodulators (e.g., kynurenine) and antimicrobial short-chain fatty acids (e.g., isovaleric acid), thus potentially increasing the risk of skin inflammation, making pathogen colonization easier, and causing damage to barrier function (Neis et al., 2015; Platten et al., 2019). In contrast, increase in abundance of homologous recombination pathway, which has association with DNA damage repair, might reflect genetic damage brought by thermal stress (Cox et al., 2000). This suggests that microbiota may rely on efficient repair mechanisms for maintenance of genomic stability. Furthermore, enrichment of metabolic pathways suggests that high temperature may trigger bacterial energy metabolism (e.g., glycolysis, TCA cycle) so as to realize rapid energy supply, or strengthen stress tolerance through synthesis of heat shock proteins (HSPs; Fleury et al., 2009). In summary, although high temperatures macroscopically promote the morphological development of the skin, they concurrently inhibit the skin microbiota’s tryptophan metabolism and the degradation of valine, leucine, and isoleucine. This leads to the impairment of microbiota-mediated skin barrier function and immune regulation in *Bufo gargarizans* tadpoles, reflecting a decoupling of morphological development from local metabolic homeostasis. Ultimately, the microbiota may cope with thermal stress by enhancing metabolic pathways and activating DNA repair.

## Data Availability

The datasets presented in this study can be found in online repositories. The names of the repository/repositories and accession number(s) can be found at: https://www.ncbi.nlm.nih.gov/, PRJNA1440655; https://www.ncbi.nlm.nih.gov/, PRJNA1440650.
