# Supplementary material for: Gut and skin microbiota of Bufo gargarizans tadpoles respond differently to temperature
Source: Front Microbiol. 2026 May 5;17:1835806. doi: 10.3389/fmicb.2026.1835806 (PMC13183613; doi:10.3389/fmicb.2026.1835806)
Supplement: Supplementary file 1 [file Data_Sheet_1.DOCX]

**Supplementary Figures and Tables**

1. **Supplementary Figures**

**
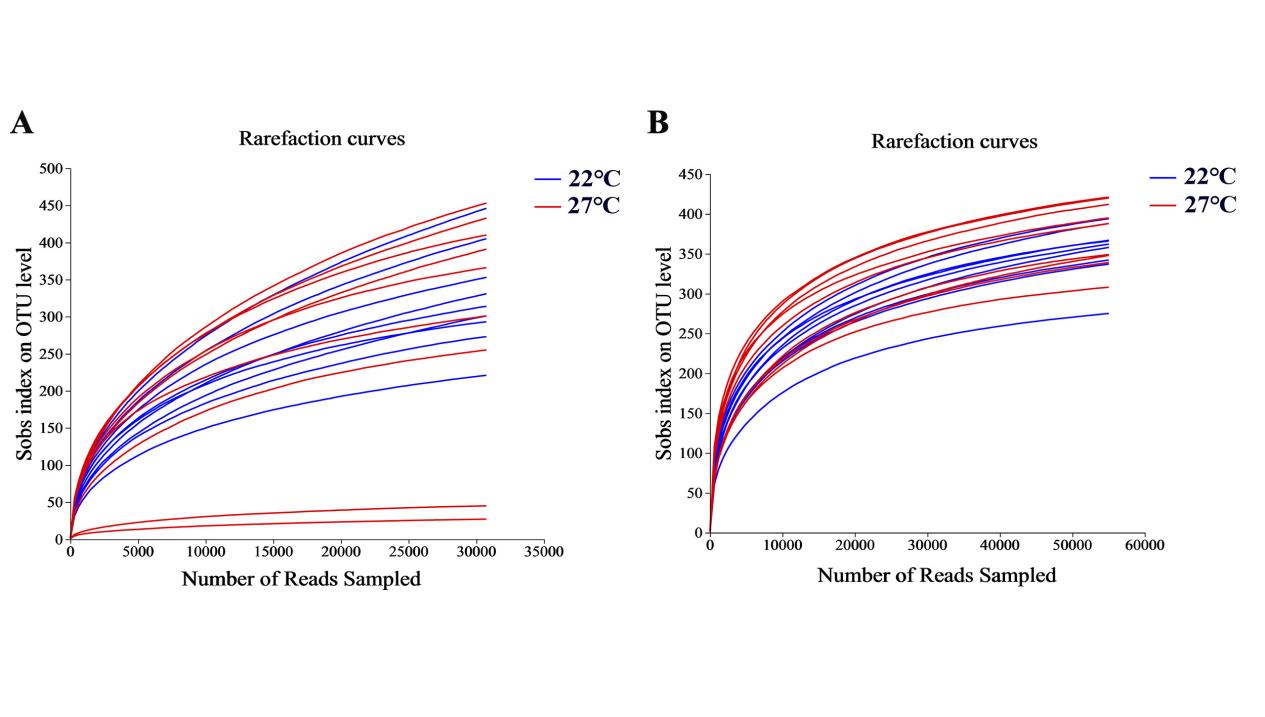
**

**Figure S1** Rarefaction curves of the gut and skin microbiota of *B. gargarizans* tadpoles under different temperature treatments. The curves illustrate the trends in species richness (Sobs index) at the OTU level with increasing sequencing depth (number of reads sampled) in the 22℃ (blue) and 27℃ (red) treatment groups. The leveling off of the curves indicates that the current sequencing depth sufficiently captures the microbial diversity of the samples. (**A**) Rarefaction curves of the gut microbiota; (**B**) Rarefaction curves of the skin microbiota

**
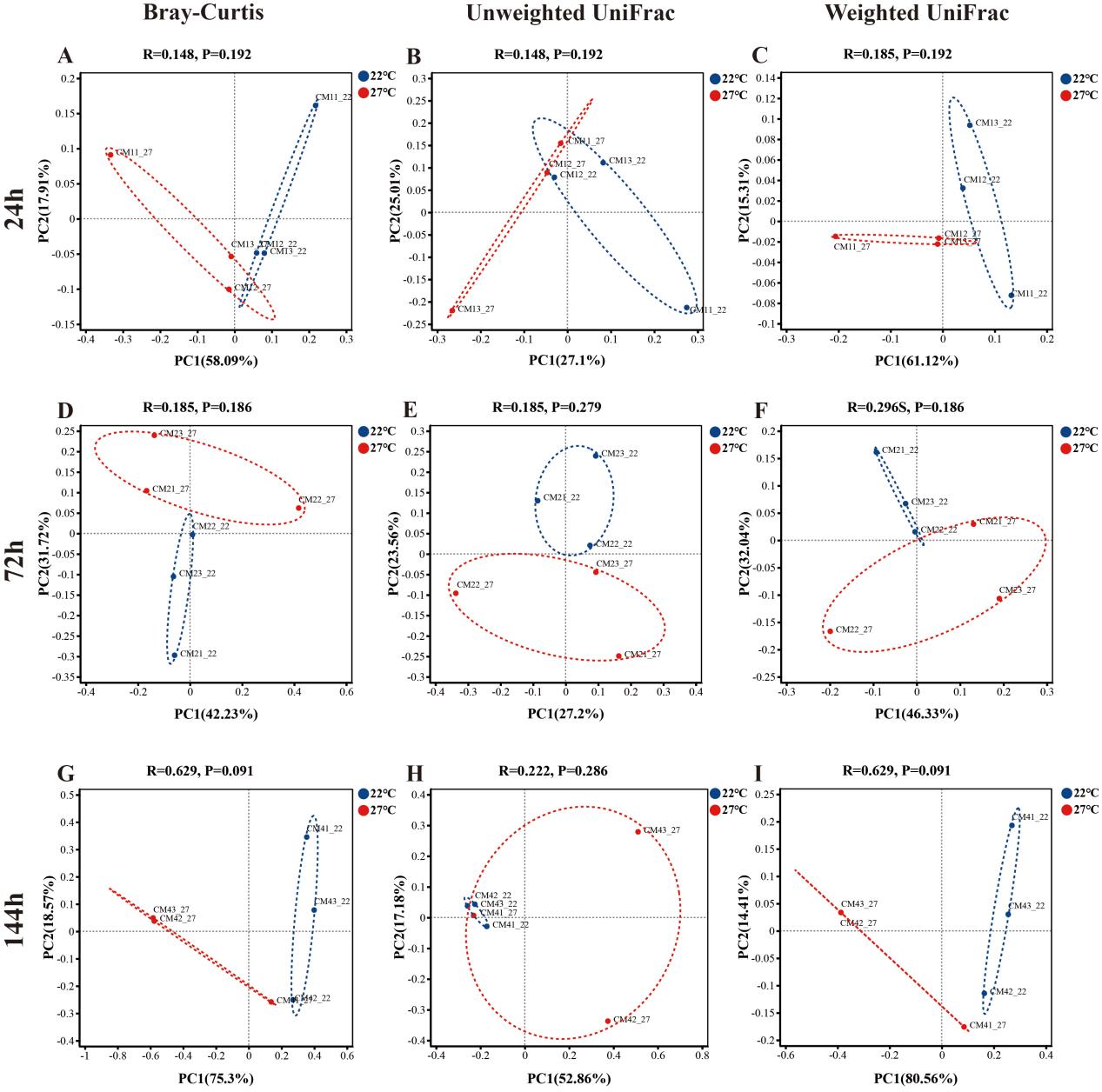
**

**Figure S2** Principal coordinate analyses plots of *B. gargarizans* gut microbiota at the 24 hour (**A-C**); 72 hour (**D-F**); and 144 hour (**G-I**) experimental time points, based on Bray-Curtis, unweighted UniFrac and weighted UniFrac dissimilarity across samples. Points are colored by temperature treatment (blue: 22℃; red: 27℃), and ellipses represent the 95% confidence interval of that treatment group. On each plot, the results of PERMANOVA models assessing the temperature effects on gut microbial community composition at that time point are displayed, including the F statistic from the model, and the FDR corrected p-values (q-value). Percentages on the axes of PCoA plots indicate the proportion of variation explained by that axis.

**
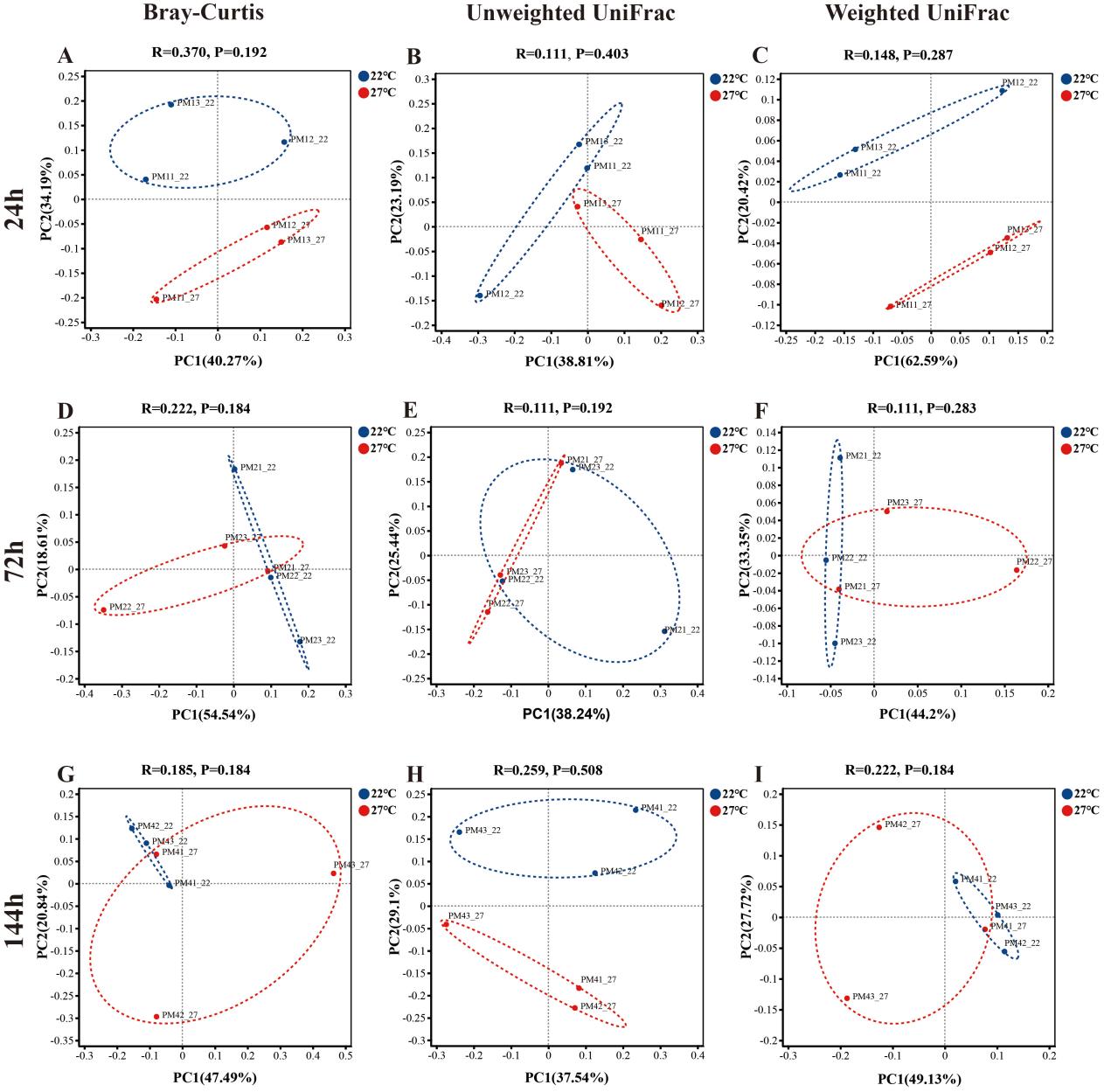
**

**Figure S3** Principal coordinate analyses plots of *B. gargarizans* skin microbiota at the 24 hours (**A-C**); 72 hours (**D-F**); and 144 hours (**G-I**) experimental time points, based on Bray-Curtis, unweighted UniFrac and weighted UniFrac dissimilarity across samples. Points are colored by temperature treatment (blue: 22℃; red: 27℃), and ellipses represent the 95% confidence interval of that treatment group. On each plot, the results of PERMANOVA models assessing the temperature effects on skin microbial community composition at that time point are displayed, including the F statistic from the model, and the FDR corrected p-values (q-value). Percentages on the axes of PCoA plots indicate the proportion of variation explained by that axis.

**
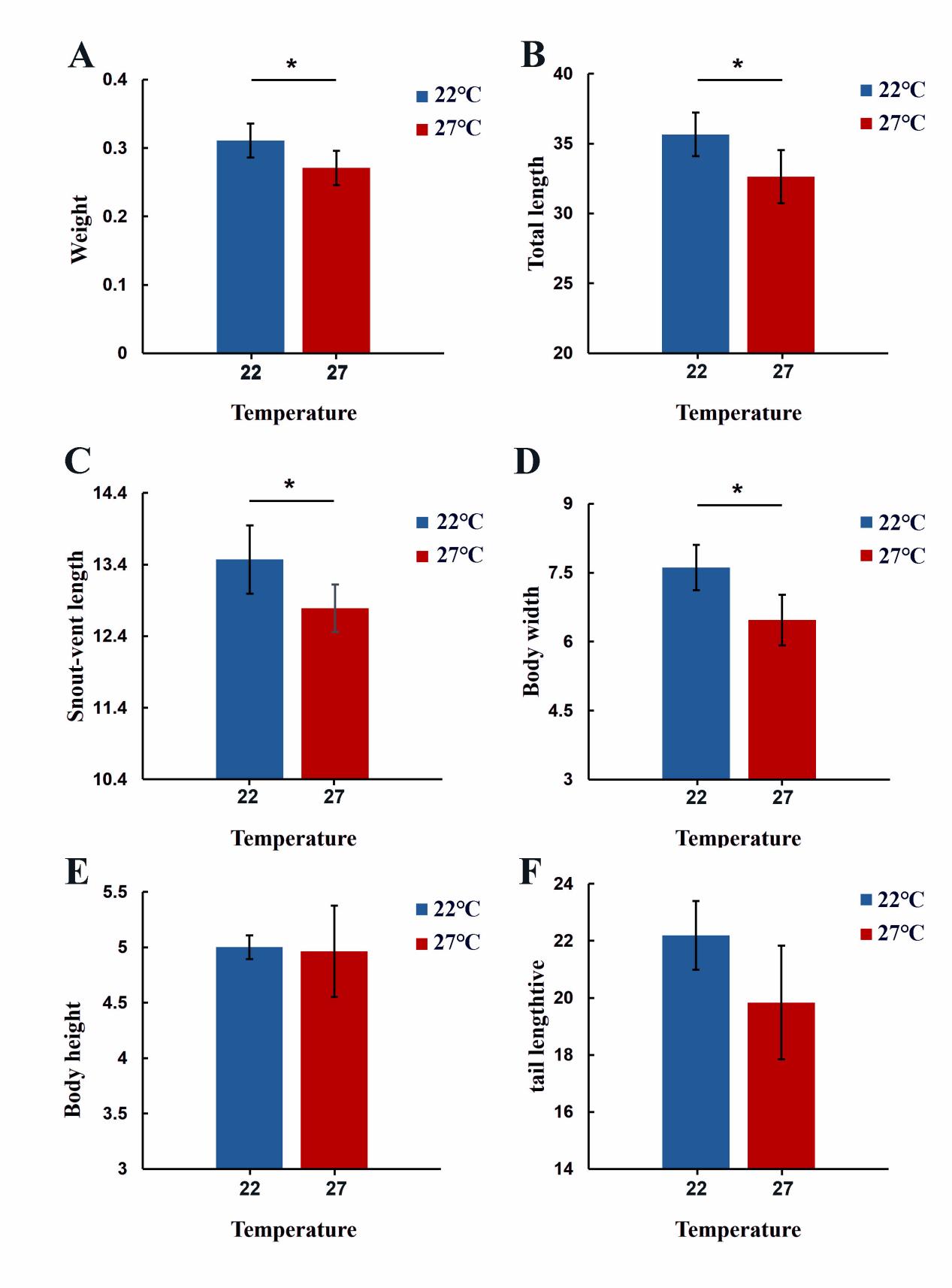
**

**Figure S4** Morphological parameters of Gs42 *B. gargarizans* tadpoles after 144 hours under two temperature treatments. (**A**) Weight; (**B**) Total length; (**C**) Snout-vent length (SVL); (**D**) Body width; (**E**) Body height; (**F**) Tail length. control group (22℃): n = 5; high temperature group (27℃): n = 4. Data met normality (Shapiro-Wilk test, p>0.05) and homogeneity of variance. Differences between groups were analyzed using independent samples t-test, with data presented as mean ± SD. ***** indicate significant differences between groups.

**
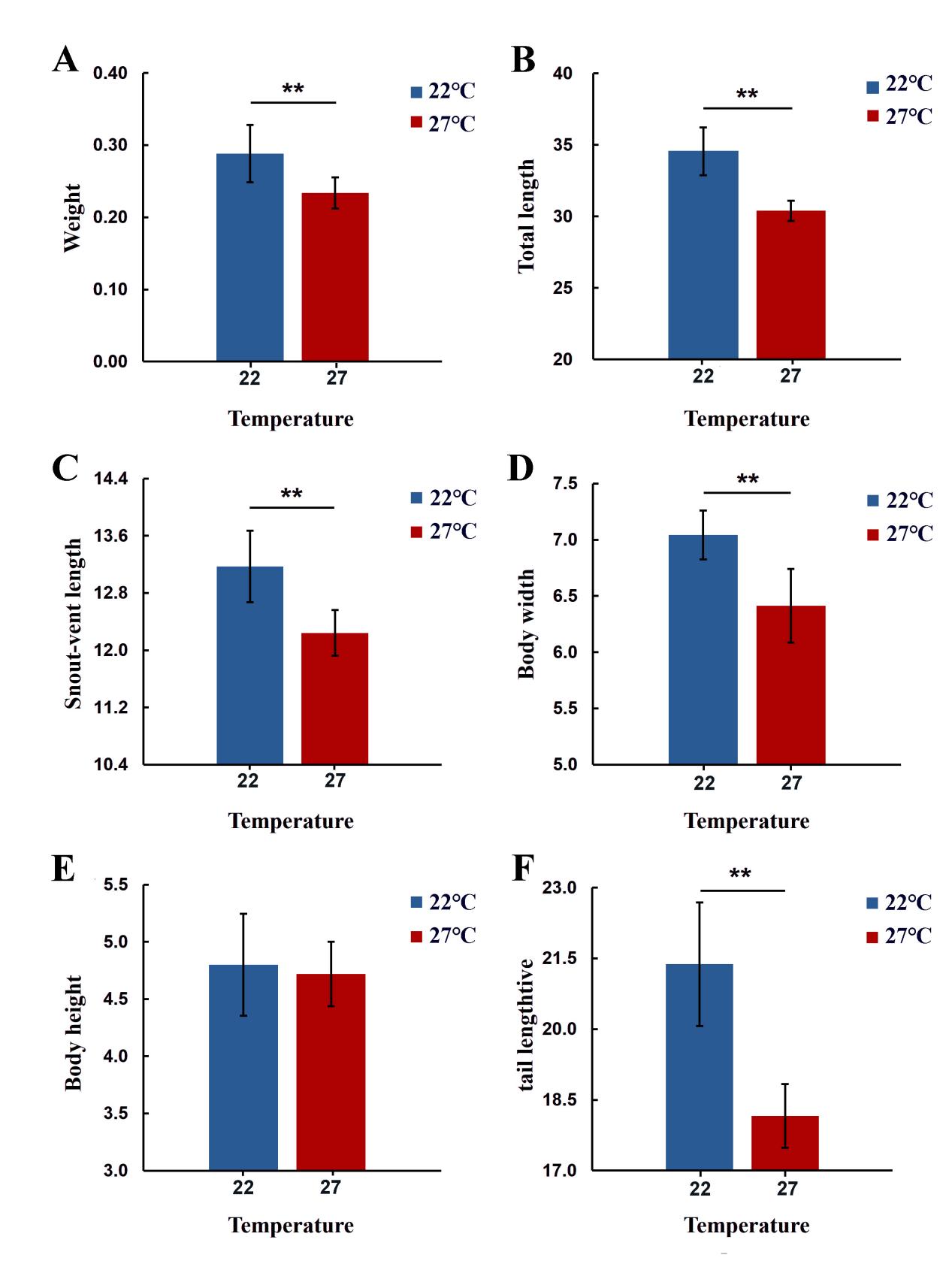
**

**Figure S5** Morphological parameters of Gs43 *B. gargarizans* tadpoles after 144 hours under two temperature treatments. (**A**) Weight; (**B**) Total length; (**C**) Snout-vent length (SVL); (**D**) Body width; (**E**) Body height; (**F**) Tail length. control group (22℃): n = 6; high temperature group (27℃): n = 10. Data met normality (Shapiro-Wilk test, p>0.05) and homogeneity of variance. Differences between groups were analyzed using independent samples t-test, with data presented as mean ± SD. ***** indicate significant differences between groups.

1. **Supplementary Tables**

**Table S1** The alpha diversity indices of the *B. gargarizans* tadpoles gut microbiota were analyzed using linear mixed models (LMMs). The F-statistic and original p-value are reported for each fixed effect. For significant effects, p-values from post hoc pairwise comparisons were adjusted using the false discovery rate (FDR) method.

| **Alpha** | **Factor** | **F statistic** | **df** | ***P* -value** |
| --- | --- | --- | --- | --- |
| ACE | temp | 0.498 | 14 | 0.492 |
|  | time | 14.349 | 14 | **0.002**** |
|  | temp*time | 0.501 | 14 | 0.491 |
| Chao | temp | 1.157 | 14 | 0.300 |
|  | time | 16.378 | 14 | **0.001**** |
|  | temp*time | 0.615 | 14 | 0.446 |
| Shannon | temp | 0.444 | 14 | 0.516 |
|  | time | 4.000 | 14 | 0.065 |
|  | temp*time | 5.156 | 14 | **0.039*** |
| Simpson | temp | 1.293 | 14 | 0.275 |
|  | time | 3.442 | 14 | 0.085 |
|  | temp*time | 6.668 | 14 | **0.022*** |
| Sobs | temp | 0.543 | 14 | 0.473 |
|  | time | 18.759 | 14 | **0.001**** |
|  | temp*time | 3.703 | 14 | 0.075 |
| Pd | temp | 0.745 | 14 | 0.402 |
|  | time | 19.170 | 14 | **0.001**** |
|  | temp*time | 3.928 | 14 | 0.067 |

**Table S2** The alpha diversity indices of the *B. gargarizans* tadpoles skin microbiota were analyzed using linear mixed models (LMMs). The F-statistic and original p-value are reported for each fixed effect. For significant effects, p-values from post hoc pairwise comparisons were adjusted using the false discovery rate (FDR) method.

| **Alpha** | **Factor** | **F statistic** | **df** | ***P*-value** |
| --- | --- | --- | --- | --- |
| ACE | temp | 0.604 | 14 | 0.45 |
|  | time | 1.015 | 14 | 0.331 |
|  | temp*time | 1.246 | 14 | 0.283 |
| Chao | temp | 0.65 | 14 | 0.434 |
|  | time | 1.641 | 14 | 0.221 |
|  | temp*time | 1.118 | 14 | 0.308 |
| Shannon | temp | 0.804 | 14 | 0.385 |
|  | time | 0 | 14 | 0.995 |
|  | temp*time | 2.259 | 14 | 0.155 |
| Simpson | temp | 0.025 | 14 | 0.876 |
|  | time | 0.037 | 14 | 0.85 |
|  | temp*time | 1.329 | 14 | 0.268 |
| Sobs | temp | 1.453 | 14 | 0.248 |
|  | time | 0.733 | 14 | 0.406 |
|  | temp*time | 1.821 | 14 | 0.199 |
| Pd | temp | 0.064 | 14 | 0.805 |
|  | time | 0.565 | 14 | 0.465 |
|  | temp*time | 9.006 | 14 | **0.010*** |

**Table S3** The effects of temperature and time on the gut and skin bacterial communities of *B. gargarizans* tadpoles were tested by PERMANOVA based on Bray-Curtis dissimilarity matrices. The model results report the F-statistic and false discovery rate (FDR)-corrected p-value (q-value) for each term.

|  | **Metric** | **Factor** | **F statistic** | **q-value** |
| --- | --- | --- | --- | --- |
|  | **bray-curtis** |  |  |  |
| **Gut microbiota** |  | temp | 2.719 | **0.032*** |
|  |  | time | 5.041 | **0.001**** |
| **Skin microbiota** |  | time | 4.525 | **0.001*** |

**Table S4** Analysis of morphological parameters in Gs42 stage *B. gargarizans* tadpoles under two temperature treatments. Data normality was verified using the Shapiro-Wilk test. An independent samples t-test was then performed for data analysis.

| Morphological Parameter | Control group | High temperature group | T-test | |
| --- | --- | --- | --- | --- |
|  | n = 5 | n = 4 | t | *P* |
| Weight | 0.311±0.025 | 0.271±0.025 | 2.367 | **0.050*** |
| Total length | 35.660±1.557 | 32.630±1.906 | 2.633 | **0.034*** |
| Snout-vent length (SVL) | 13.471±0.478 | 12.791±0.332 | 2.404 | **0.047*** |
| Body width | 7.613±0.493 | 6.471±0.550 | 3.285 | **0.013*** |
| Body height | 5.001±0.106 | 4.963±0.412 | 0.178 | 0.869 |
| Tail length | 22.189±1.204 | 19.839±1.996 | 2.200 | 0.064 |

**Table S5** Analysis of morphological parameters in Gs43 stage *B. gargarizans* tadpoles under two temperature treatments. Data normality was verified using the Shapiro-Wilk test. An independent samples t-test was then performed for data analysis.

| Morphological Parameter | Control group | High temperature group | T-test | |
| --- | --- | --- | --- | --- |
|  | n = 6 | n = 10 | t | *P* |
| Weight | 0.288 ± 0.040 | 0.234 ± 0.022 | 3.579 | **0.003**** |
| Total length | 34.551 ± 1.673 | 30.400 ± 0.704 | 7.000 | **0.000**** |
| Snout-vent length (SVL) | 13.172 ± 0.500 | 12.242 ± 0.320 | 4.575 | **0.000**** |
| Body width | 7.043 ± 0.217 | 6.414 ± 0.328 | 4.153 | **0.001**** |
| Body height | 4.800 ± 0.445 | 4.718 ± 0.282 | 0.455 | 0.656 |
| Tail length | 21.378 ± 1.311 | 18.158 ± 0.677 | 6.543 | **0.000**** |

1. **Supplementary Methods**

All PCR reactions were conducted in triplicate using a total volume of 20 μL reaction system containing 4 μL 5 × FastPfu Buffer, 2 μL 2.5 mM dNTPs, 0.8 μL each primer (5 μM), 0.4 μL FastPfu Polymerase, and 10 ng template DNA, with ddH_2_O added to a final volume of 20 μL. The PCR reaction was performed under the following thermocycling parameters: initial denaturation at 95℃ for 3 min, followed by 29 cycles of denaturation at 95℃ for 30 s, annealing at 55℃ for 30 s, and extension at 72℃ for 30 s, with a final extension at 72℃ for 10 min.
